# Factores asociados con la letalidad por neumonía en una unidad de atención del paciente geriátrico agudo: una cohorte retrospectiva

**DOI:** 10.7705/biomedica.5244

**Published:** 2020-12-11

**Authors:** Diana C. Quintero-González, José Mauricio Ocampo, Carlos A. Reyes-Ortiz

**Affiliations:** 1 Departamento de Medicina interna, Universidad Libre, seccional Cali, Cali, Colombia Universidad Libre Departamento de Medicina interna Universidad Libre Cali Colombia; 2 Universidad del Valle, Facultad de Salud, Departamento de Medicina Familiar, Cali, Colombia Universidad del Valle Universidad del Valle Facultad de Salud Departamento de Medicina Familiar Cali Colombia; 3 Grupo Interinstitucional de Medicina Interna, Departamento de Medicina Interna, Facultad Ciencias de la Salud, Universidad Libre, Cali, Colombia Universidad Libre Departamento de Medicina Interna Facultad Ciencias de la Salud Universidad Libre Cali Colombia; 4 Institute of Public Health, College of Pharmacy and Pharmaceutical Sciences, Florida A & M University, Tallahassee, FL, USA Florida A & M University TallahasseeFL USA

**Keywords:** neumonía, anciano, mortalidad, radiografía, nitrógeno de la urea sanguínea, evaluación de la discapacidad, Pneumonia, aged, mortality, radiography, blood urea nitrogen, disability evaluation

## Abstract

**Introducción.:**

La neumonía es la principal causa de muerte por infección en el mundo y afecta principalmente a la población de adultos mayores.

**Objetivo.:**

Determinar los factores de riesgo asociados con la letalidad a 30 días en pacientes con neumonía en una unidad de atención del paciente geriátrico agudo.

**Materiales y métodos.:**

Se hizo un estudio observacional y analítico de cohorte retrospectiva. Se incluyeron 114 pacientes de 60 años o más hospitalizados en una unidad de atención del paciente geriátrico agudo con diagnóstico de neumonía. El resultado primario fue la letalidad a 30 días. Se hicieron análisis de regresión log-binomial bivariado y multivariable para explorar la relación entre las variables independientes y el resultado primario.

**Resultados.:**

La letalidad a 30 días fue de 26,3 % y la edad media de 84,45 ± 7,37 años. El 54,4 % de los participantes era de sexo masculino. En el análisis multivariable se encontró que una edad de 90 años o más (riesgo relativo, RR=1,62; IC_95_%: 1,05-2,68; p=0,04), el compromiso multilobar (RR=1,92; IC_95_%: 1,12-3,32; p=0,02), el° nitrógeno ureico elevado (mediana≥22,5; RR=3,93; IC_95_%: 1,67-9,25; p<0,01), y un puntaje de cero en la escala de Lawton al ingreso (RR=3,20; IC_95_% 1,05-9,78; p=0,04) fueron factores predictores independientes de la letalidad a 30 días en adultos mayores con neumonía.

**Conclusión.:**

En los pacientes adultos mayores hospitalizados por neumonía en una unidad de atención del paciente geriátrico agudo la edad muy avanzada, la presencia de compromiso multilobar, la dependencia en el estado funcional y los niveles de nitrógeno ureico elevados fueron los principales factores de riesgo de letalidad a corto plazo.

## Abstract (en)

**Introduction:**

Pneumonia is the leading cause of death due to infection in the world, mainly affecting the older adult population.

**Objective:**

To determine the risk factors associated with lethality at 30 days in patients with pneumonia in an acute care for the elderly unit.

**Materials and methods:**

We conducted an observational, analytical retrospective cohort study including 114 patients aged 60 years and older hospitalized in an acute care geriatric unit with a diagnosis of pneumonia. The primary outcome was lethality at 30 days. Bivariate and multivariable log-binomial regression analyzes were performed to explore the relationship between independent variables and the primary outcome.

**Results:**

The 30-day lethality was 26.3% and the mean age was 84.45 ± 7.37 years; 54.4% of participants were men. In the multivariable analysis, age ≥90 years (Relative Risk, RR=1.62, 95% CI: 1.05-2.68, p=0.04), multilobar commitment (RR=1.92, 95% CI:1.12-3.32, p=0.02), elevated urea nitrogen (≥22.5 the median; RR=3.93, 95% CI:1.67-9.25, p<0.01), and a score of zero in the Lawton index at admission (RR=3.20, 95% CI:1.05-9.78, p=0.04) were independent predictors of 30-day lethality from pneumonia.

**Conclusion:**

In older adults hospitalized for pneumonia in an acute care geriatric unit, advanced age, the presence of multilobar commitment, dependency in functional status, and elevated ureic nitrogen levels were the main predictors of short-term lethality risk.

La neumonía es un proceso inflamatorio del parénquima pulmonar de tipo infeccioso que puede ocurrir cuando la virulencia del agente patógeno sobrepasa las defensas del huésped ^1^^,^^2^. Los adultos mayores son uno de los grupos etarios más susceptibles de desarrollar la neumonía ^(^^3^. Según la Organización Panamericana de la Salud (OPS), adulto mayor se define como una persona de 60 años o más ^4^. La población de adultos mayores en los servicios de urgencias ha aumentado, lo cual se debe al incremento uniforme y acelerado de este grupo en todo el mundo. En Europa se espera que para el 2060 la población de 65 años o más aumente del 18 al 28 % ^5^. Una situación similar se evidencia en Colombia, donde el porcentaje de mujeres y hombres adultos mayores aumentó de 7,3 y 6,7 % en 1985 hasta 13,8 y 11,5 % en el 2020, respectivamente ^6^.

A nivel mundial, la incidencia de neumonía adquirida en la comunidad tiene forma de U, pues afecta principalmente a los menores de 5 años y a los mayores de 65 ^3^. En los países europeos, la incidencia de neumonía en la comunidad puede ser 10,8 veces mayor en las personas de 85 años o más comparada con personas de 50 a 64 años ^7^. En los Estados Unidos se estima que anualmente hay 1,3 millones de casos nuevos de neumonía ^8^; además, se ha demostrado que la tasa de incidencia de la neumonía (847 casos por cada 100.000 personas/año) es superior a la de las enfermedades no transmisibles frecuentes como el infarto de miocardio y la enfermedad cerebrovascular ^9^. En Colombia, un estudio del 2009 encontró que la tasa de incidencia de neumonía en pacientes ambulatorios y hospitalizados de 50 años o mayores fue de 336 y 327 casos por cada 100.000 personas/año, respectivamente ^10^. Además, la incidencia de neumonía en pacientes hospitalizados aumentó de 326 casos por cada 100.000 personas/año en el grupo de 65 a 74 años de edad a 4.636 casos en personas de 85 años o mayores ^10^.

Por otro lado, la neumonía se cataloga como la principal causa de muerte de origen infeccioso en el mundo ^11^. En los Estados Unidos se ha reportado que la letalidad en pacientes hospitalizados es dos veces mayor en aquellos de 75 años o mayores que en los de 65 a 74 años (10,6 % *Vs.* 4,9 %) ^12^. Un estudio observacional realizado en seis países latinoamericanos encontró que la letalidad media en pacientes de 50 años o mayores hospitalizados por neumonía fue de 17,7 %, en tanto que en Colombia fue del 11 % en el 2009 ^10^. Además, en el país, la letalidad fue mayor en personas de 65 a 74 años y en las de 85 años o más (9,5 % y 13,6 %, respectivamente) ^10^. Asimismo, según las estadísticas del Departamento Administrativo Nacional de Estadística (DANE), las infecciones respiratorias agudas constituyeron el 4,23 % de las causas de muerte entre el 2016 y el 2017, ocupando el quinto lugar ^13^. Además, se ha estimado que la letalidad hospitalaria de los adultos mayores en general puede llegar a ser del 19 % ^14^.

Las unidades de atención del paciente geriátrico agudo se han diseñado para prevenir la declinación funcional y las complicaciones por yatrogenia en el adulto mayor por medio de un modelo de atención integral ^15^. Aunque su efectividad ha sido demostrada por la disminución de las caídas, el delirio, la declinación funcional y la duración de la hospitalización, no se ha descrito si su impacto en la tasa de letalidad es similar ^16^.

De hecho, la neumonía se ha descrito como la primera causa de muerte en estas unidades, con tasas elevadas que oscilan entre el 37,5 y el 40 % ^17^. A pesar de las opciones de tratamiento, la vacunación y la disponibilidad de pruebas diagnósticas, la letalidad por neumonía en los adultos mayores sigue siendo alta. En los últimos 10 años los factores que se han asociado con la letalidad por esta condición son la edad, un índice de comorbilidad de Charlson elevado, comorbilidades como la enfermedad pulmonar obstructiva crónica (EPOC), la enfermedad cerebrovascular, la fractura de cadera, las neoplasias sólidas, la malnutrición, el estado mental alterado, la hipotensión, el grado de dependencia, el compromiso bilateral y el derrame pleural ^18^^-^^24^.

En Colombia, son pocos los estudios que han investigado los factores asociados con la letalidad en la población de adultos mayores. El presente estudio pretende contribuir a cerrar esta brecha en el conocimiento, determinando los factores asociados con la letalidad a 30 días en pacientes con neumonía en una unidad de atención del paciente geriátrico agudo.

## Materiales y métodos

### Diseño del estudio y recolección de la información

Se hizo un estudio observacional de cohorte retrospectiva de 114 pacientes con edad igual o mayor a 60 años y diagnóstico de neumonía hospitalizados en una unidad de atención del paciente geriátrico agudo entre marzo de 2011 y agosto de 2015.

Para ingresar a la unidad, los pacientes debían tener una edad igual o mayor a 60 años y cumplir con uno o más de los siguientes criterios: múltiples comorbilidades (dos o más), condición funcional previa de dependencia grave o total, hospitalizaciones frecuentes (dos o más al mes), demencia previa o delirio al ingreso, presencia de dos o más síndromes geriátricos (por ejemplo, fragilidad, úlceras por presión o caídas), índice de masa corporal menor de 20 kg/m^2^, red de apoyo social insuficiente, proceder de instituciones geriátricas, o tener más de 80 años con enfermedad aguda.

Para el diagnóstico de neumonía se consideraron los criterios publicados en la guía colombiana de 2013. Los pacientes debían cumplir con, al menos, dos de los siguientes signos y síntomas: temperatura mayor o igual a 38,3 °C, tos productiva, dolor torácico, disnea o crepitación en la auscultación, además de una nueva opacidad en la radiografía de tórax ^1^. Se excluyeron los pacientes con antecedente de infección por el virus de inmunodeficiencia humana (VIH), tuberculosis activa y aquellos con variables clínicas no incluidas en la historia clínica. Los datos analizados ya estaban recolectados y se seleccionó esta cohorte de forma retrospectiva.

### Variables de estudio

*Resultado.* Consistió en la letalidad a 30 días posterior al diagnóstico de neumonía en pacientes hospitalizados en una unidad de atención del paciente geriátrico agudo. La información de letalidad se recolectó de las historias clínicas y fue corroborada con las estadísticas vitales suministradas por la Secretaría de Salud Departamental del Valle del Cauca mediante los certificados de defunción hasta septiembre del 2015. Los códigos CIE-10 descritos como causa directa de muerte fueron: A419, septicemia no especificada; J159, neumonía bacteriana no especificada; J960, insuficiencia respiratoria aguda, y J969, insuficiencia respiratoria no especificada.

*Variables independientes.* Las variables sociodemográficas incluían la edad, el sexo y la escala de valoración sociofamiliar de Gijón (menor a 8 puntos: sin riesgo social). Las variables clínicas fueron la hospitalización en días; el índice de comorbilidad de Charlson (sin comorbilidad: 1 punto, comorbilidad baja: 2 puntos, comorbilidad alta: 3 o más puntos) ^25^; el número de medicamentos al ingreso, y la presencia de las siguientes comorbilidades: hipertensión, enfermedad cerebrovascular, diabetes mellitus, dislipidemia, EPOC, enfermedad renal crónica, fibrilación auricular, demencia, hipotiroidismo, trastorno de la deglución, insuficiencia cardiaca, epilepsia, cáncer y tabaquismo.

En cuanto al examen físico, se incluyó la frecuencia cardiaca (latidos por minuto), la frecuencia respiratoria (respiraciones por minuto), la hipotensión sistólica definida como una tensión arterial sistólica menor o igual a 90 mm Hg, y la hipoxemia definida como oximetría menor o igual a 90 % o presión arterial de oxígeno menor o igual a 60 mm Hg en gases arteriales.

En cuanto a las variables de laboratorio y de imagenología, se incluyeron la presencia de derrame pleural o compromiso multilobar (dos o más lóbulos pulmonares) en la radiografía de tórax, leucocitos (células/mm3), hemoglobina (g/dl), albúmina (mg/dl), creatinina (mg/dl), nitrógeno ureico (mg/dl) empleado como variable numérica pero también categorizado como BUN *(blood urea nitrigen)* elevado de acuerdo a la mediana (≥22,5 *Vs.* <22,5) en la construcción de riesgo relativo, sodio (mg/dl), y proteína C reactiva (PCR; mg/dl).

El resultado de las variables de laboratorio correspondió a su primera medición en el momento del ingreso del paciente. Además, se calculó la escala CURB-65, la cual describe las siguientes variables: confusión, nitrógeno ureico mayor o igual a 20 mg/dl, frecuencia respiratoria mayor o igual a 30 respiraciones por minuto, tensión arterial sistólica menor o igual a 90 mm Hg y edad mayor o igual a 65 años ^26^; cada variable suma un punto en la escala, o sea que a mayor número de puntos mayor la gravedad del cuadro. Con base en estos parámetros los pacientes se clasificaron en tres grupos de riesgo: grupo 1 (0 a 1 punto), grupo 2 (2 puntos) y grupo 3 (3 o más puntos).

Para la evaluación de la capacidad funcional en las actividades básicas de la vida diaria se tomó el índice de Barthel al ingreso ^27^, considerando a un paciente como dependiente si obtenía un puntaje menor o igual a 60 puntos. También se incluyó la evaluación de las actividades instrumentales de la vida diaria mediante la escala de Lawton y Brody en el momento de ingreso ^28^, categorizando a los pacientes como totalmente dependientes (0 puntos) o no (1 a 8 puntos). Por último, se documentó la presencia de delirio y deterioro cognitivo a través de la escala del *Confusion Assessment Method* (CAM) ^29^ y la escala Mini-Mental, respectivamente ^30^, así como el esquema antibiótico y el número de días de tratamiento.

### Análisis estadístico

Se empleó estadística descriptiva para resumir las características de la población. Las variables cuantitativas se expresaron en medidas de tendencia central y dispersión. Para evaluar la distribución de los datos se usó la prueba de Kolmogórov-Smirnov y se asumió como significativos los valores de p<0,05. Las variables cualitativas se resumieron como proporciones y se presentaron en tablas de frecuencia. La significación estadística de las asociaciones en las variables numéricas se determinó con la prueba U de Mann Whitney, y la de las variables dicotómicas con ji al cuadrado o la prueba de Fisher según correspondiera; para rechazar las hipótesis nulas se asumieron como significativos valores de p<0,05. También se hizo un análisis de sensibilidad para evaluar si los excluidos eran significativamente diferentes de los participantes.

Para explorar la presencia de asociaciones entre la variable de resultado (letalidad a 30 días) y las variables de exposición, se hicieron los análisis de regresión log binomial bivariado y multivariable. Para ello se usó el *Genmod Procedure* en SAS™, el cual utiliza la distribución de Poisson para estimar los riesgos relativos (RR) con intervalos de confianza (IC) del 95 %.

Para ajustar los posibles factores predictores se utilizó un modelo de regresión multivariable. Para la selección de dicho modelo se usó el criterio modificado de Greenland ^31^. Inicialmente, se seleccionaron aquellas variables con p<0,20 asociados tanto con la variable dependiente (letalidad) como con la principal variable independiente (elegimos el BUN, ya que en otro estudio la función renal baja se encontró asociada con una mayor mortalidad) ^32^. Las que produjeron una variación significativa igual o mayor del 10 % en la asociación entre la variable dependiente y la principal, se mantuvieron en el modelo multivariable. Para el diagnóstico de este modelo se usaron parámetros tales como la convergencia, la multicolinealidad y la sobredispersión. La bondad del ajuste se evaluó con la prueba de la desviación, la cual mostró muy buen ajuste (grados de libertad=109; valor=54,66; p=0,99).

Además, se calculó el riesgo relativo de letalidad como la razón de riesgo en los expuestos (a/[a+b] son los pacientes que tienen uno o más factores de riesgo: edad≥90 años, hipoxemia, dependencia total en la escala de Lawton, BUN alto y compromiso multilobar) dividido por el riesgo en los no expuestos (c/[c+d] pacientes sin algún factor de riesgo) y se calcularon los IC_95%_. Los análisis estadísticos se realizaron en los programas Stata™ (Stata Corp, 2011, Stata 12 Base Reference Manual, College Station, TX, USA) y SAS™, versión 9.4 para Windows (SAS Institute, Inc., Cary, NC).

### Consideraciones éticas

El protocolo del estudio primario, del cual se recolectó la información para este trabajo de investigación, contó con el consentimiento informado y las precauciones necesarias para garantizar la privacidad de los pacientes (la protección de los datos anonimizados y el manejo dado por el equipo investigador). El estudio fue aprobado por el Comité de Ética y Científico de la Universidad Libre y de la Clínica Universitaria Rafael Uribe Uribe.

## Resultados

**Figura 1:**
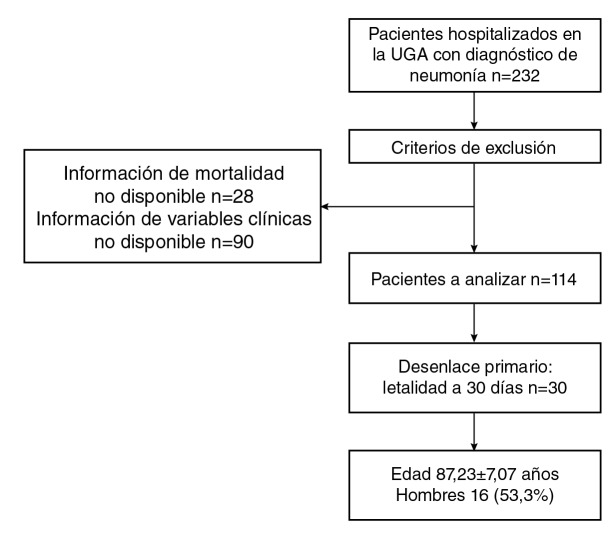
Pacientes de la unidad de atención del paciente geriatrico agudo con diagnóstico de neumonía entre el 2011 y el 2015

De 232 pacientes con diagnóstico principal o secundario de neumonía, 28 fueron excluidos por no tener información disponible sobre mortalidad y en 90, porque la mayoría de las variables clínicas relacionadas con el desenlace no pudieron ser verificadas en la historia clínica (figura 1). Comparados con los pacientes que se incluyeron en el estudio (n=114), los pacientes excluidos (n=118) tenían más dependencia en cuanto a su estado funcional (con un puntaje en la escala de Lawton significativamente más bajo: p=0,03, y en el índice de Barthel: p<0,01), pero no tenían diferencias significativas de edad, sexo, comorbilidades, estado mental (MMSE) ni presencia de delirio. El resultado primario, es decir, la letalidad en los 30 días posteriores al diagnóstico, se presentó en 30 pacientes. La media de tiempo entre el diagnóstico y el desenlace fatal fue de 16 ± 7,8 días.

Las características sociodemográficas, funcionales, clínicas y de los exámenes de laboratorio se resumen en los cuadros 1 y 2. Se encontró una media de edad superior en los pacientes con el resultado primario (87,2 años *Vs.* 83,5 años). No hubo diferencias significativas en la relación hombre-mujer, el estado sociofamiliar, la duración de la hospitalización en días, el índice de comorbilidad de Charlson y el número de medicamentos, entre otras descritas en el cuadro 1. La evaluación de la capacidad funcional en el momento de ingreso demostró que los pacientes del grupo donde hubo muertes tenían una mayor proporción de dependencia total según el índice de Barthel (93,3 % *Vs.* 63,1 %; p<0,01). Este hallazgo fue similar en la escala de Lawton y Brody, en el que el 90,0 % del grupo que registró muertes tenía dependencia total (puntaje de cero) comparado con el 56,0 % en el grupo de quienes no la tenían (p<0,01). El porcentaje de delirio evaluado con la escala del *Confusión Assessment Method* fue de 46,7 % *Vs.* 36,9 % (p=0,35).

**Cuadro 1:** Características sociodemográficas y clínicas de la población de estudio

Características	General	Letalidad a 30 días		p
	n=114 n (%)	Sí (n=30) n (%)	No (n=84) n (%)	
Edad^*^	84,45 (7,37)	87,23 (7,07)	83,45 (7,25)	0,02
Edad >90	28 (24,6)	12 (40,0)	16 (19,0)	0,02
Sexo				
Masculino	62 (54,39)	16 (53,33)	46 (54,76)	0,89
Femenino	52 (45,61)	14 (46,67)	38 (45,24)	
Escala de Gijón				
Sin riesgo social^0^	98 (85,96)	28 (93,33)	70 (83,33)	
Con riesgo social	16 (14,04)	2 (6,67)	14 (16,67)	0,23 t
Hospitalización en días^**^	12 (9-16)	12,5 (10-17)	12 (9-15)	0,37
Índice de Charlson^**^	3 (2-4)	3 (2-5)	3 (2-4)	0,48
Número de medicamentos al ingreso^**^	5 (3-8)	5 (3-8)	5 (3-7,5)	0,92
Antecedentes patológicos				
Hipertensión	73 (64,04)	19 (63,33)	54 (64,29)	0,93
Tabaquismo	40 (35,71)	9 (31,03)	31 (37,35)	0,54
EPOC	38 (33,33)	8 (26,67)	30 (35,71)	0,37
Diabetes mellitus	28 (24,56)	3 (10,00)	25 (29,76)	0,03
ECV	22 (19,30)	8 (26,67)	14 (16,67)	0,23
Enfermedad renal crónica	19 (16,67)	6 (20,00)	13 (15,48)	0,57
Demencia	18 (15,79)	8 (26,67)	10 (11,90)	0,08 t
Hipotiroidismo	15 (13,16)	2 (6,67)	13 (15,48)	0,35 t
Insuficiencia cardíaca	13 (11,40)	2 (6,67)	11 (13,10)	0,51 t
Dislipidemia	10 (8,77)	3 (10,00)	7 (8,33)	0,72t
Trastorno de deglución	7 (6,14)	3 (10,00)	4 (4,76)	0,38 t
Cáncer	7 (6,14)	3 (10,00)	4 (4,76)	0,38 t
Neumonía previa	6 (5,26)	1 (3,33)	5 (5,95)	1,00 t
Epilepsia	6 (5,26)	4 (13,33)	2 (2,38)	0,04 t
Fibrilación auricular	5 (4,39)	2 (6,67)	3 (3,57)	0,61 t
Variables clínicas				
Hipotensión sistólica	15 (13,16)	5 (16,67)	0 (11,90)	0,54 t
Hipoxemia	54 (47,37)	22 (73,33)	32 (38,10)	< 0,01
Frecuencia cardíaca^**^	84 (77-98)	81 (80-90)	86,5 (75-99,5)	0,51
Frecuencia respiratoria^**^	19 (16-24)	18 (16-26)	20 (17-23)	0,73
CURB-65^**^	2 (1-3)	3 (2-3)	2 (1-3)	< 0,01
CURB-65 n (%)				
Neumonía leve^*^	70 (61,40)	11 (36,67)	59 (70,24)	< 0,01
Neumonía grave	44 (38,60)	19 (63,33)	25 (29,76)	
Tratamiento n (%)				
Recibió antibiótico	111 (97,37)	30 (100,00)	81 (96,43)	0,57 t
Duración (días)^**^	10 (8-11)	10 (7-11)	9 (8-10)	0,70

* Media y desviación estándar

^**^ Mediana y rangos intercuartílicos; test exacto de Fisher ^0^ Sin riesgo social <8 puntos

* Neumonía leve <3 puntos

EPOC: enfermedad pulmonar obstructiva crónica; ECV: enfermedad cerebrovascular

**Cuadro 2:** Características funcionales y mentales, y resultados en exámenes paraclínicos de la población de estudio

Características	General	Letalidad a 30 días		p
	n (%)	Sí (n=30) n (%)	No (n=84) n (%)	
Indice de Barthel al ingreso				
Independiente	33 (28,95)	2 (6,67)	31 (36,90)	<0,01
Dependiente^♦^	81 (71,05)	28 (93,33)	53 (63,10)	<0,01
Escala de Lawton al ingreso				
Sin dependencia total	40 (35,09)	3 (10,0)	37 (44,0)	<0,01
Con dependencia total^┴^	74 (64,91)	27 (90,0)	47 (56,0)	<0,01
MiniMental				
Deterioro cognitivo leve ^θ^	63 (55,26)	13 (43,33)	50 (59,52)	0,13
Deterioro cognitivo grave	51 (44,74)	17 (56,67)	34 (40,48)	0,13
Delirio (CAM)	45 (39,47)	14 (46,67)	31 (36,90)	0,35
Variables paraclinicas				
Compromiso multilob	6 (5,26)	4 (13,33)	2 (2,38)	0,04 t
Derrame pleural	29 (25,44)	8 (26,67)	21 (25,00)	0,86
Leucocitos^**^	11.440 (8.540-15.230)	10.510 (8.730-13.250)	11.690 (8.525 - 15.355)	0,49
Hemoglobina^**^	11,65 (9,6-12,7)	10,6 (9,5-12,4)	11,85 (9,95-13,1)	0,26
Albumina^**^	2,97 (2,63-3,3)	2,72 (2,34-3,09)	3,01 (2,75-3,37)	0,01
Creatinina^**^	1,01 (0,74-1,41)	1,04 (0,75-1,26)	1,01 (0,73-1,48)	0,94
Nitrogeno ureico^**^	22,5 (17-34)	34 (25-54)	20 (15-26,5)	< 0,01
Sodio^**^	140 (137-144,4)	142,63 (137-149,1)	139,83 (137-142)	0,1
Proteina C reactiva^**^	73,66 (23,59-140)	85,06 (61,23 - 129,7)	65,99 (19,53 - 148,83)	0,20

* Media y desviación estándar

*^*^ Mediana y rangos intercuartílicos; test exacto de Fisher

^♦^ Dependiente: ≤ 60 puntos

^┴^ Con dependencia total: 0 puntos

^θ^ Deterioro cognitivo leve: >23 puntos;

CAM: *Confusion Assessment Method*

En el examen físico, la hipoxemia se presentó en el 73,3 % de los pacientes del grupo donde hubo muertes (p=0,001). Según los resultados de laboratorio, la mediana y el rango intercuartílico (RIC) de nitrógeno ureico fue de 34 mg/dl en el grupo en el que se registraron muertes a los 30 días (RIC: 25 - 54 mg/dl) comparado con 20 mg/dl (RIC: 15 - 26,5mg/dl) (p<0,01) en el otro grupo; asimismo, el 83,3 % del grupo en el que hubo muertes tenían un BUN≥22,5 mg/dl comparado con el 38,1 % de los sobrevivientes (p<0,01). No hubo diferencias en el número de leucocitos al ingreso o en los reactantes de fase aguda como la proteína C reactiva. El compromiso multilobar en la radiografía de tórax fue mayor en el grupo en el que hubo muertes (13,3 % *Vs.* 2,4%, p=0,04). La evaluación de la gravedad según la escala CURB65 demostró un porcentaje más alto de pacientes del grupo 3 en el grupo en el que hubo muertes (66,3 % *Vs.* 29,8 %; p<0,01), lo que indica una mayor gravedad del cuadro. Los antibióticos más utilizados fueron los inhibidores de betalactamasas (ampicilina más sulbactam y piperacilina más tazobactam): 76,7 % en el grupo en el que hubo muertes *Vs.* 73,8 % en los que sobrevivieron los primeros 30 días. No hubo diferencias significativas en el número de días de tratamiento (p=0,7).

**Figura 2:**
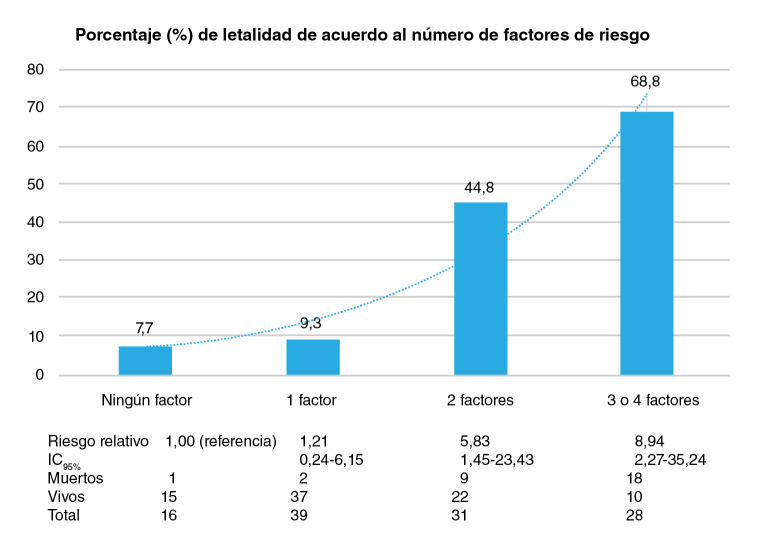
Porcentaje de letalidad debido a neumonía según el número de factores de riesgo

En el análisis bivariado las siguientes variables tuvieron una asociación significativa con la letalidad: edad ≥90 años (RR=2,05; IC_95%_: 1,13-3,70, índice de Barthel (RR=5,05; IC_95%_: 1,63-15,64), escala de Lawton y Brody (RR=4,86; IC_95%_: 1,57-15,05), epilepsia (RR=2,77; IC_95%_: 1,09-36,44), hipoxemia (RR=3,05; IC_95%_: 1,49-5,34), compromiso multilobar (RR=2,77; IC_95%_: 1,43-5,34), nitrógeno ureico (RR=1,02; IC_95%_: 1,01-1,03), BUN≥22,5 mg/ dl (R°R=5,00; IC_95%_: 2,06-12,14), y CURB65 (RR=2°,75; IC_95%_: 1,45-5,21). Los resultados completos del análisis bivariado se resumen en el cuadro 3. En el análisis de regresión multivariable, la edad de 90 años o más (p=0,04), un puntaje cero en la escala de Lawton y Brody categorizado como dependencia total (p=0,04), la presencia de compromiso multilobar (p=0,02) y el valor del nitrógeno ureico elevado (p<0,01) se asociaron de manera independiente con la letalidad a los 30 días (cuadro 4). En la figura 2 se observa un aumento de la letalidad (p<0,001) y del riesgo relativo con un mayor número de factores de riesgo (edad >90 años, compromiso multilobar, puntaje de cero en la escala de Lawton, BUN alto), especialmente con la presencia de, por lo menos, dos factores a la vez. 

**Cuadro 3:** Regresiones en el análisis log-binomial bivariado de los factores asociados con la letalidad a 30 días

Características	RR	IC_95%_	p
Edad^*^	1,05	1,02-1,09	<0,01
Edad ≥90	2,05	1,13-3,70	0,02
Escala de Gijon	0,44	0,11-1,66	0,22
Indice de Barthel al ingreso^*^	0,98	0,96-0,99	<0,01
Indice de Barthel al ingreso^•^	5,05	1,63-15,64	<0,01
Escala de Lawton al ingreso^*^	0,56	0,25-0,89	0,01
Escala de Lawton al ingreso^┴^	4,86	1,57-15,05	<0,01
Minimental	1,61	0,87-3,00	0,13
Diabetes mellitus	0,34	0,11-1,04	0,06
Demencia	1,94	1,03-3,65	0,04
Epilepsia	2,77	1,43-5,34	<0,01
Hipoxemia	3,05	1,49-6,28	<0,01
Compromiso multilobar	2,77	1,43-5,34	<0,01
Albumina^*^	0,46	0,27-0,80	<0,01
Nitrogeno ureico^*^	1,02	1,01-1,03	<0,01
Nitrogeno ureico≥22,5 mg/dl	5,00	2,06-12,14	<0,01
CURB-65^*^	1,46	1,16-1,84	<0,01
CURB-65 ^Ǿ^	2,75	1,45-5,21	<0,01

* Variables numericas

^•^ Dependiente: ≤ 60 puntos

^┴^ Con dependencia total: 0 puntos

^Ǿ^ CURB-65≥3 puntos

RR: riesgo relativo; IC: intervalo de confianza

**Cuadro 4:** Regresión log-binomial múltiple de los factores asociados con la letalidad a 30 días

Características	RR	IC_95%_	p
Escala de Lawton al ingreso: 0 puntos	3,20	1,05-9,78	0,04
Edad ≥90	1,62	1,05-2,68	0,04
Multilobar	1,92	1,12-3,32	0,02
Nitrogeno ureico ≥22,5 mg/dl	3,93	1,67-9,25	< 0,01

RR: riesgo relativo; IC: intervalo de confianza

La bondad del ajuste se evaluó con la prueba de desviación (p=0,99).

Los factores de riesgo incluyen la edad igual o mayor de 90 años, el compromiso multilobar, puntaje de cero en la escala de Lawton, y BUN alto. Hubo un aumento significativo en las muertes (p<0,001) a medida que el número de factores de riesgo aumentaba (n=114).

## Discusión

En este estudio de pacientes adultos mayores con neumonía hospitalizados en una unidad de atención del paciente geriátrico agudo se encontró que la presencia de BUN elevado (≥22,5 mg/dl), la dependencia total en las actividades instrumentales según la escala de Lawton y Brody al ingreso, el compromiso multilobar y la edad avanzada (≥90 años) fueron los principales factores predictivos de letalidad a los 30 días.

El BUN es un parámetro de laboratorio importante en la evaluación del paciente con neumonía; su elevación esta mediada por la hipoperfusión renal que genera la activación del sistema renina-angiotensina-aldosterona, la hormona arginina-vasopresina y el sistema simpático, generando una reabsorción de sodio, agua y urea ^33^. Es una de las variables de las escalas CURB65 y PSI, y aunque se ha descrito su peso en la asociación con la letalidad, su relevancia en el paciente adulto mayor es controversial. A pesar de que la descripción de los niveles de BUN es frecuente en los estudios, la mayoría no ha demostrado una asociación estadísticamente independiente con la letalidad a corto plazo ^19^^-^^21^^,^^23^^,^^24^.

Sin embargo, los resultados de dos cohortes, una retrospectiva procedente de Singapur y otra prospectiva de España, publicados en el 2018 respaldaban la asociación con esta variable. En el primer estudio se evaluaron 1.902 pacientes según su grupo etario y se encontró que en participantes entre los 65 y 84 años los niveles de BUN superiores a 31 mg/dl tenían una asociación con la mortalidad a 30 días (OR=2,0; IC_95%_: 1,3-3,1), hecho que se replicó, además, en la población mayor de 84 años ^34^. En el segundo estudio, de Uranga, *et al.,* en participantes con una media de edad de 69 años, de los cuales el 33 % era mayor de 80 años, se demostró que un BUN de más de 30 mg/dl era una variable con asociación estadística con la mortalidad a un año a partir del episodio de neumonía (HR=1,55; IC_95%_: 1,07-2,24) ^35^. En la población del presente estudio se validaron estos resultados al demostrar que un BUN de 22,5 mg/dl o más en el momento de ingreso se relacionaba con un aumento mayor a tres veces del riesgo de muerte a los 30 días en pacientes adultos mayores con neumonía (RR=3,93; IC_95%_: 1,67-9,25).

La discapacidad como la imposibilidad total o parcial de participar en las actividades de la vida diaria se atribuye al efecto negativo de la interacción entre el paciente anciano y el medio ambiente. La discapacidad está asociada con el incremento en el costo de la asistencia sanitaria ^36^, la disminución de la calidad de vida ^37^ y la mortalidad. En el 2016 un estudio retrospectivo en 1.834 pacientes entre los 60 y los 84 años demostró que la discapacidad aumentaba dos veces el riesgo de muerte por cualquier causa ^38^. En el presente estudio la segunda variable con mayor magnitud de asociación con el resultado primario fue la pérdida de la funcionalidad en las actividades instrumentales medida por la escala de Lawton y Brody al ingreso, donde un puntaje de 0 indicaba un riesgo tres veces mayor de letalidad a los 30 días después de un episodio de neumonía.

Este hecho se documentó previamente en el 2014 en un estudio de Calle, *et al.,* en 456 pacientes de 75 años de edad o más, en quienes se demostró que conservar el dominio funcional (>5 puntos en la escala de Lawton y Brody) se relacionaba de manera independiente con una menor letalidad a corto plazo, configurándose como un factor protector (OR=0,09; IC_95%_: 0,010,81) ^21^. Ello se explicaría por la tendencia acelerada de envejecimiento en la población y el incremento en la expectativa de vida asociado con la pérdida paulatina de la funcionalidad, que empieza a cobrar relevancia con las limitaciones para llevar a cabo las actividades instrumentales. En el análisis bivariado en la cohorte del presente estudio, un puntaje menor de 60 (paciente dependiente) en el IBi fue un factor de riesgo de letalidad a los 30 días (RR=5,05; IC_95%_: 1,63-15,64), sin embargo, su significación estadística no se mantuvo en el análisis multivariable. Este hecho contrasta con los resultados obtenidos en estudios previos, en los que una dependencia funcional moderada o grave en las actividades básicas se asoció con un riesgo tres veces mayor de letalidad a corto plazo ^22^, y un índice de Barthel menor de 40 puntos se asoció con la letalidad hospitalaria a tres meses y a un año, con un OR de 9,45, 6,84 y 19,55, respectivamente, determinando el impacto de la discapacidad a corto y largo plazo ^39^.

El compromiso de dos o más lóbulos en la radiografía de tórax se ha descrito tradicionalmente como un factor que contribuye a la hospitalización y el riesgo de muerte por neumonía, aunque con un peso secundario en la magnitud del riesgo comparado con los parámetros fisiológicos. Un metaanálisis del 2013 en el que se evaluó el impacto de la neumonía multilobar en 11.456 pacientes de 22 estudios, la mayoría prospectivos, demostró que este hallazgo radiográfico es un factor de riesgo de letalidad, con un RR de 2,57 ^40^. El presente estudio corrobora este hallazgo en la población adulta mayor, siendo el compromiso multilobar la tercera variable estadísticamente relacionada con la letalidad a corto plazo, con un RR de 1,92. Resultados similares se obtuvieron en una cohorte del 2014 ^21^.

En el siglo XIX, antes de la era antimicrobiana, la neumonía fue descrita por Sir William Osler como una enfermedad fatal en el paciente anciano. En la actualidad, a pesar del tratamiento efectivo y el diagnóstico temprano, la tasa de letalidad en la neumonía sigue siendo alta. Según la edad y la gravedad de la enfermedad, puede variar entre el 4,9 y el 48 % ^41^. En la presente cohorte la tasa de letalidad a 30 días fue de 26,3 %, lo que la sitúa en el límite superior del rango descrito en la literatura (6,8 a 28,7 %). Este alto porcentaje puede explicarse por la edad cronológica avanzada, de 84,5 años en promedio, superior a la de otros estudios ^22^^,^^39^. En estudios previos se ha demostrado el impacto de la edad en la letalidad, incluso en cohortes de pacientes muy ancianos, y se ha estimado el aumento del riesgo de muerte en adultos con edades de 80 años o más (OR=2,10; IC_95%_: 1,40-3,15) ^35^, de 85 o más (OR=3,01; IC_95%_: 1,71-5,30) ^22^ y de 90 años o más (OR=3,10; IC_95%_: 1,31-7,36) ^21^. Este hecho se corroboró en nuestra población al demostrar que una edad de 90 años o más confiere un RR de 1,62 asociado con la letalidad a 30 días.

Como se ha descrito en la literatura especializada, la enfermedad cerebrovascular, la enfermedad pulmonar, el tabaquismo, la enfermedad renal crónica y la hipertensión son comorbilidades frecuentes ^2^, aunque sin significación estadística en términos de asociación con la letalidad a 30 días. Varios autores han encontrado que la alteración del estado de conciencia constituye un predictor de la letalidad ^20^^,^^22^ y, aunque en el presente estudio no hubo diferencias en esta variable y los pacientes que presentaron el resultado primario tenían un 10 % más de alteración del estado de conciencia, posiblemente el tamaño de la muestra y el delirio como criterio de ingreso en la unidad geriátrica de agudos atenuaron el resultado. La hipoxemia en el adulto mayor es una de las variables clínicas relevantes en el diagnóstico de neumonía y, aunque en un estudio fue descrita como un factor de riesgo de letalidad, con un OR de 2,7 ^34^, en nuestro estudio no se demostró la asociación independiente con la muerte.

Se ha demostrado que los sistemas de puntuación establecidos para determinar la gravedad de la neumonía y el sitio de manejo ideal, el CURB65 y *Pneumonia Severity Index* (PSI) no tienen la suficiente precisión para clasificar al paciente adulto mayor ^2^. El alto valor que da el PSI a la edad y las comorbilidades suele sobreestimar el riesgo, en tanto que la ausencia de la carga de enfermedad como variable en la escala CURB65 puede subestimar el riesgo en los pacientes adultos mayores, que pueden descompensarse de manera significativa, incluso cuando se trata de neumonías catalogadas como leves en dicha escala. Un estudio del 2010 que incluyó tres grupos etarios (18-64, 65-84, y ≥85 años) corroboró este hallazgo al demostrar un poder discriminativo decreciente del PSI y la CURB-65 con respecto a las categorías de edad avanzada, con un área bajo la curva en la CURB65 de 0,80, 0,73 y 0,6 en cada grupo, respectivamente ^42^. En nuestra población, un puntaje en la escala CURB65 mayor o igual a 3 implicaba un mayor riesgo de letalidad a corto plazo, con un RR de 2,75, aunque esta asociación no fue relevante en el análisis multivariado.

Por último, los resultados demuestran la importancia de abordar la neumonía en el adulto mayor (incluso desde el ingreso en el servicio de urgencias) con un enfoque multidimensional; en nuestra cohorte una variable sociodemográfica, una de laboratorio, una de imagenología y una funcional explicaron la letalidad a corto plazo. El modelo presentado evidenció que las variables descritas tuvieron un efecto aditivo en el riesgo de letalidad a 30 días, por lo que tener dos o tres o más variables incrementó más de 5 y 8 veces la posibilidad de un desenlace adverso, respectivamente. El objetivo con estos resultados es cambiar la perspectiva en la atención del adulto mayor. Una adecuada clasificación del paciente en cuanto al riesgo permite tomar decisiones acertadas sobre el manejo integral, lo que se traduce en una reducción de la letalidad de una enfermedad frecuente que cobra miles de víctimas cada año alrededor del mundo.

La fortaleza del presente estudio reside en que los casos de neumonía habían sido confirmados por radiología en una población con criterios de selección estrictos que permitieron determinar los factores con impacto en la letalidad a 30 días en pacientes hospitalizados en una unidad de atención del paciente geriátrico agudo, población con altas tasas de morbimortalidad que poco se ha considerado en los estudios. Además, la cohorte analizada estaba internada en un servicio especializado de hospitalización geriátrica con funciones académicas y asistenciales a cargo de un equipo interdisciplinario. Además, para garantizar la calidad de la información cada escala aplicada y cada dato obtenido fue responsabilidad de profesionales entrenados durante años en el área específica (médicos y profesionales de rehabilitación, psicología y trabajo social).

Una de las limitaciones fue la información secundaria y el posible sesgo de selección, ya que se excluyeron pacientes con mayor dependencia funcional y aquellos cuyos datos estaban incompletos, pues esto afecta la generalización de los resultados. Asimismo, el tamaño de la muestra pudo atenuar la magnitud de la asociación de algunas variables; además, la condición retrospectiva puede incrementar el error de medición, pues no hay control sobre las fuentes de información y es difícil ajustar por otros factores predictores durante el análisis. Tampoco se pudo incluir el dato de la procedencia (hogar familiar *Vs.* hogar geriátrico) debido a la información incompleta.

Es importante destacar que se trata de una cohorte de adultos mayores con una media de edad avanzada, de 84 años, y con al menos una comorbilidad, lo cual limita la validez externa de los resultados en otros pacientes que cumplan con las características planteadas de la población objetivo del presente estudio. Estas limitaciones motivan la realización de estudios de cohorte prospectiva y preferiblemente multicéntricos que permitan establecer otros factores potencialmente modificables que afectan la supervivencia de los pacientes.

En los pacientes ancianos hospitalizados por neumonía en esta unidad de atención del paciente geriátrico agudo, la edad muy avanzada (≥90 años), la presencia de compromiso multilobar, la dependencia total en el estado funcional (según la escala de Lawton y Brody al ingreso) y los niveles de nitrógeno ureico elevados fueron los principales predictores de riesgo de letalidad a corto plazo. Estas variables deben ser evaluadas en el momento de ingreso de pacientes adultos mayores con neumonía para caracterizar mejor el riesgo y definir intervenciones que favorezcan su funcionalidad y supervivencia.
